# Description of characteristics and outcomes of a cohort of patients with severe and enduring eating disorders (SE-ED)

**DOI:** 10.1186/s40337-021-00492-8

**Published:** 2021-10-20

**Authors:** Ana Piñar-Gutiérrez, Elena Dios-Fuentes, Pablo Remón-Ruiz, Diego Del Can-Sánchez, Antonio Vázquez-Morejón, Marta López-Narbona, Javier Dastis-Rodríguez de Guzmán, Eva Venegas-Moreno, Alfonso Soto-Moreno

**Affiliations:** 1 Endocrinology and Nutrition Department, Virgen del Rocío University Hospital, Sevilla, Spain; 2 Mental Health Department, Virgen del Rocío University Hospital, Sevilla, Spain; 3 Centro de Diagnóstico Y Tratamiento, Planta Baja: Endocrinología Y Nutrición, Av Manuel Siurot sn, CP 41013 Sevilla, Andalucía, Spain

**Keywords:** Eating disorders, Anorexia nervosa, Bulimia nervosa, Osteoporosis, Hypogonadotropic hypogonadism

## Abstract

**Objective:**

To describe the characteristics of the patients, as well as the treatment outcomes for the people treated in an Endocrinology and Nutrition unit with a diagnosis of SE-ED (> 7 years evolution despite evidence-based conventional treatment).

**Methods:**

A descriptive observational study was conducted. Patients with a diagnosis of SE-ED (anorexia nervosa and bulimia nervosa) treated in the Endocrinology and Nutrition service of the Virgen del Rocío University Hospital between 2014 and 2019 were included.

**Results:**

67 patients were contacted and accepted to participate in the study. 95.5% were women. 67.2% were diagnosed with AN (anorexia nervosa) and 32.8% with BN (bulimia nervosa). Their median ages (years) at the onset of symptoms, beginning of follow-up and at present were 17, 32 and 42.5 respectively. Their median time of follow-up was 9 years. 73.1% had mental comorbitidy and AN patients had more osteoporosis (48.9% vs 22.7%, *p* = 0.04) and hypogonadotropic hypogonadism (31.1% vs. 4.5%, *p* = 0.014).

**Discussion:**

The SE-ED patients in our sample began treatment years after the onset of symptoms, which may have led to their chronification. This emphasizes the importance of an early diagnosis in eating disorders. They presented with a high rate of physical complications and mental comorbidity. In the current sample, it was determined that patients with AN presented with higher rates of osteoporosis and hypogonadotropic hypogonadism than patients with BN.

**Level of evidence:**

Level III: Evidence obtained from well-designed cohort or case–control analytic studies.

**Plain English summary:**

At present, the criteria for severe and enduring eating disorders (SE-ED) are not sufficiently clearly defined. It has been calculated that approximately 20% of patients with anorexia nervosa (AN) and 10% of patients with bulimia nervosa (BN) suffer a chronification. We evaluated the characteristics of the patients, as well as the treatment outcomes for the people treated in an Endocrinology and Nutrition unit with a diagnosis of SE-ED (which was made based on an evolution greater than 7 years despite conventional treatment). The SE-ED patients in our sample began treatment years after the onset of symptoms, which may have led to their chronification. They presented with a high rate of physical complications and mental comorbidity. In the current sample, it was determined that patients with AN presented with higher rates of osteoporosis (health condition that weakens bones, making them fragile and more likely to break) and hypogonadotropic hypogonadism (illness in which testes or ovaries produce little or no sex hormones due to a problem in the pituitary gland) than patients with BN.

## Introduction

Among eating disorders (ED), resistance to treatment is equivalent to chronicity, that is, it is a permanent aspect of the disease despite having performed different kinds of evidence-based therapies [1].

At present, the criteria for severe and enduring ED (SE-ED) are not sufficiently clearly defined. Current criteria are based on the duration of the disorder, together with unsuccessful previous attempts at conventional treatment. Nowadays, when conventional treatment fails over a period of between 7 and 10 years, the disease is considered to be chronic, and a different therapeutic approach is recommended to increase the chances of successful treatment [2, 3]. Even so, there is no consensus on the number of failed treatment attempts needed before reaching a diagnosis of treatment resistance. There is also no consensus as to whether patients who have not received treatment or those who have received it but have intermediate results should be included in the category of SE-ED [3, 4]. In bulimia nervosa (BN) SE-ED should be considered when there is no response to either evidence-based psychotherapy or selective serotonin reuptake inhibitors (SSRI) treatment. Regarding anorexia nervosa (AN), resistance to treatment should be suspected when there is a repeated failure to maintain 85% of the ideal weight after multiple psychotherapeutic and intensive feedback treatment attempts in specialized programs, both on an outpatient basis and in hospitalization [5]. On the other hand, severity is described by the Diagnostic and Statistical Manual of Mental Disorders (DSM-5) in terms of body mass index (BMI), although clinical symptoms, functional disability and need for supervision are also included [6].

Currently, the evidence on treatment options for SE-ED is very limited. Although improvement in nutritional status is very important, the main focus should be related to quality of life and social adaptation and to a lesser degree on the significant reduction of ED symptoms. This is because overly demanding treatment goals can promote endurance and with detrimental effects, including increased risk of suicide [1, 2, 7]. Long-term poor nutritional status and disordered eating behavior impair cognitive structure and functions. Because of this, patients with SE-ED may benefit from innovative new treatment options, such as deep brain stimulations designed to change the factors that maintain the disorder [8].

Despite all this, at present there are few studies that evaluate the results of SE-ED treatment, despite the fact that there are studies in which it has been calculated that approximately 20% of patients with AN and 10% of patients with BN suffer a chronification [9–11]. Furthermore, in the few studies where the results have been compared in patients with SE-ED vs patients with non SE-ED, a worse prognosis has been reported in patients with SE-ED. Specifically SE-ED patients present a greater number of complications and higher mortality [12].

The objective of our study is to evaluate the characteristics, as well as the treatment outcomes (mental and physical) for the patients treated in our unit with a diagnosis of SE-ED.

## Methods

A descriptive observational study was carried out in which patients with a diagnosis of SE-ED and treated in the Endocrinology and Nutrition service of the Virgen del Rocío University Hospital between 2014 and 2019 were included. We operationalized SE-ED as a disorder lasting 7 or more years [2, 3, 7] despite conventional treatment in specialized programs (outpatient, inpatient and day hospital). All patients included gave their consent to participate in this study.

Endocrinology follow-up was performed on an outpatient basis, and patients could be admitted to the hospital during their evolution. During hospitalization, patients were cared for jointly by the Endocrinology Unit and the Mental Health Unit. The criteria for admission to the Endocrinology ward were: failure of outpatient treatment; weight loss greater than 25% compared to the previous one in less than 6 months or 10% in one month; a heart rate < 50 bpm or blood pressure < 90/60 (80/50 in children) and / or associated symptoms; a potassium < 3 mEq/L or Na < 130 mg / dL, and / or changes in the electrocardiogram; the presence of severe hypoproteinemia and-/- or edema in the lower limbs; and/or the presence of persistent hypoglycemia and/or hypertransaminasemia. When the reason for admission is due to a mental illness, patients are admitted to the Mental Health hospital floor with nutritional support from us.

Regarding Mental Health Unit follow-up, it could be performed on an outpatient and/or day patient basis. After hospitalization, all patient treatments were performed on a day patient basis, followed by outpatient treatment if there was evolutionary improvement.

The variables collected are listed in Table 1.

**Table 1:** List of variables collected

Sex
Type of eating disorder (AN or BN according to the criteria of the DSM-5 Manual^6^)
Follow-up carried out in Endocrinology and Mental Health consultations
Follow-up time in Endocrinology’s Unit
Current age
Age of onset of eating disorder symptoms
Age of beginning of follow-up in Endocrinology’s Unit
Current BMI
Minimum BMI
Percentage of BMI recovered ((current BMI-minimum BMI) / minimum BMI)
Presence of mental comorbidity *Personality disorders* *Depression* *Anxiety* *Bipolar disorder* *Obsessive–compulsive disorder*
Abusive intake of alcohol or other toxins—depending on DSM-5 criteria for Substance Use Disorders^6^
History of suicide behavior disorder
Presence of physical complications *Osteoporosis -according to WHO criteria-* *Hypogonadotropic hypogonadism* *Chronic kidney disease -glomerular filtration* < *60 ml/min/1.73m2 for more than three months- or nephrocalcinosis diagnosed by ultrasound* *Digestive alterations such as chronic constipation or gastroesophageal reflux* *Ionic disorders (-high or low sodium levels, potassium, magnesium, phosphorus and / or calcium in the blood-)*
Number of admissions per person to the Endocrinology ward
Days of admission to the Endocrinology ward
Death of the patient

Statistical analysis was carried out using Statistical Package for Social Science (SPSS®) 25 version for Windows (IBM Corporation, New York, USA). The descriptive analysis was carried out by obtaining the median and the quartiles of the quantitative variables (expressed as P50(P25-P75)) and the frequency for the qualitative variables (expressed as n(%)), both in the total sample and in AN and BN subgroups. For the inferential analysis between the two subgroups, a Chi-square test was performed for qualitative variables and the median test for independent samples for quantitative variables. P-values below 0.05 were considered statistically significant.

## Results

### Patients’ characteristics

A total of 67 patients were contacted to take part in the study and 67(100%) accepted to be included. 64 (95.5%) were women. Regarding the distribution of ED type, 45 (67.2%) were diagnosed with AN and 22 (32.8%) with BN.

The proportion of patients that underwent each form of treatment (outpatient, day patient and inpatient) in their clinical evolution is represented in Table 2. 39 (58.2%) patients underwent hospitalization, 30(66.7%) in the AN group and 9 (40.9%) in the BN group.

**Table 2:** Basis treatment performed in patients with severe and enduring eating disorders in an Endocrinology and Nutrition unit during 2014 and 2019

Basis treatment	Total	AN	BN	*p* value
Outpatient only	12 (17.9%)	5 (11.1%)	7 (31.8%)	0.068
Outpatient + Day patient	16 (23.9%)	10 (22.2%)	6 (27.3%)	
Outpatient + Day patient + Inpatient	39 (58.2%)	30 (66.7%)	9 (40.9%)	

The follow-up in both consultations was higher than 80% if all patients who had some kind of follow-up, whether regular or irregular, are taken into account. The results regarding the median time of follow-up in consultations were 9 (6–14) years in the total sample, 11 (7–14.5) years in the group with AN and 7.5(5.75–13.25) years in the BN group.

**Fig. 1:**
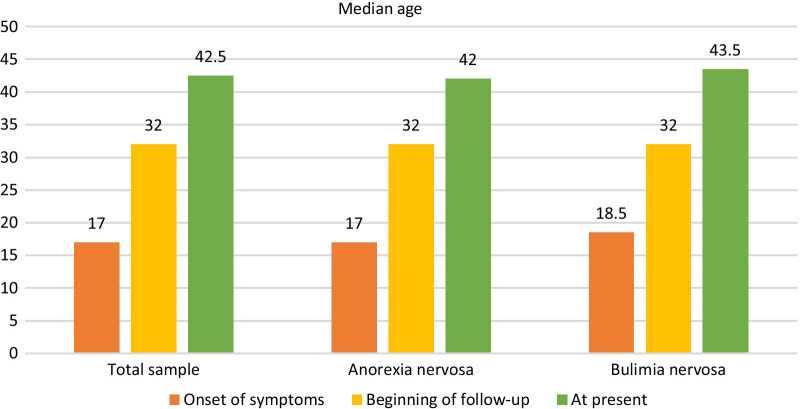
Median age of patients with chronic eating disorders seen between 2014 and 2019 at the onset of symptoms, at the beginning of follow-up, and at present

Figure 1 shows the median age (years) of the patients at the onset of symptoms, at the beginning of follow-up by the Endocrinology and Nutrition Unit and at present, both in the total sample and in the subgroups of patients with AN and BN. Regarding the total sample, the median ages were 17, 32 and 42.5 years old, respectively.

### Health outcomes

Table 3 shows the results obtained when studying the physical and mental complications that arose throughout the follow-up of the patients, as well as the need for admission to the Endocrinology and Nutrition floor and BMIs obtained. The results are shown both for the total sample and for the subgroups of AN and BN.

**Table 3:** Minimum and current BMI, as well as percentage of BMI recovered and complications arising during the follow-up of patients with severe and enduring eating disorders in an Endocrinology and Nutrition unit during 2014 and 2019

Variable	Total	AN	BN	*p* value
**Minimum BMI (kg/m**^**2**^**)**	**16.2 (14.6–18.7)**	**15.75 (14–16.4)**	**19.4 (16.9–22.8)**	**0.00**
**Current BMI (kg/m**^**2**^**)**	**19 (17.1–20.8)**	**18 (16.5–19.7)**	**22.3 (19–25.3)**	**0.04**
Recovered BMI (%)	12.74 (7.1–26)	14.6 (7.6–25.2)	10.4 (2.5–26.7)	0.64
Mental comorbidity	49 (73.1%)	33 (73.3%)	16 (72.7%)	0.958
Suicide behavior disorder	9 (13.4%)	5 (11.1%)	4 (18.2%)	0.425
Substance use disorder	10 (14.9%)	5 (11.1%)	5 (22.7%)	0.210
CKD/nephrocalcinosis	4 (6%)	2 (4.4%)	2 (9.1%)	0.451
Digestive alterations *Chronic constipation* *Gastroesophageal reflux*	19 (28.4%) *13 (19.7%)* *12 (17.9%)*	14 (31.1%) *9 (20%)* *8 (17.8%)*	5 (22.7%) *4 (18.2%)* *4 (18.2%)*	0.475 0.860 0.968
Ionic disorders	13 (19.4%)	8 (17.8%)	5 (22.7%)	0.630
**Osteoporosis**	**27 (40.3%)**	**22 (48.9%)**	**5 (22.7%)**	**0.04**
**Hypogonadotropic hypogonadism**	**15 (22.4%)**	**14 (31.1%)**	**1 (4.5%)**	**0.014**
Endocrinology Ward admissions	1 (0–2)	1 (0–3.5)	0 (0–1.25)	0.261
Days of admission to Endocrinology ward	13.5 (9.7–19.7)	14,5 (9.7–23.5)	12 (8.5–13.7)	0.225
Exitus	1 (1.5%)	1(2.2%)	0(0%)	

The minimum BMI was 16.2 (14.6–18.7)kg/m2, with significant differences between patients with AN and BN (15.75 (14–16.4) vs. 19.4 (16.9–22, 8), *p* = 0.000). The current BMI was 19 (17.1–20.8) kg/m2, with significant differences between the subgroups of AN and BN (17.8 (16.5–19.7) vs. 22.3 (19–25.3), *p* = 0.04). Despite this, no significant differences were found when comparing the percentage of recovered BMI in both groups (14.6 (7.6–25.2)% vs. 10, 4 (2.5–26.7)%, *p* = 0.640).

49 patients (73.1%) presented with mental comorbidity, 9(13.4%) presented with a history of attempted autolysis, and 10 (14.9%) presented with abusive consumption of toxic substances. Significant differences were found between both subgroups in terms of the proportion of osteoporosis (48.9% vs. 22.7%, *p* = 0.04) and hypogonadotropic hypogonadism (31.1% vs. 4.5%, *p* = 0.014). The median number of admissions per person was 1 (0–2) and the median days of each admission was 13.5 (9.7–19.7). 1 (1.5%) patient died during follow-up.

## Discussion

Our study is characterized by evaluating the health results obtained in patients with ED, focusing exclusively on those who have become chronic. At present, the published studies in which these patients are referred to are usually studies of years of follow-up in which the predisposing factors for greater chronicity or mortality are evaluated, in some of them comparing results between acute and chronic patients [13–15]. This may be due to the lack of established criteria on the definition of chronicity in these disorders, which makes the study of these patients, and therefore the improvement in their care, difficult.

As all patients presenting with SE-ED were included in the study, the data are representative of our population of people presenting with this group of diseases. The data obtained regarding the sex of the patients accords with those recorded in the medical literature, with female:male ratios described as ranging from 6:1 to 10:1 [16]. Regarding the type of eating disorder, in our sample AN prevailed over BN, according to current scientific evidence reported about SE-ED [7].

Regarding these patients’ follow-up, there are no studies evaluating their follow-up rates or abandonment of consultations rates, but given their characteristics we believe that the results obtained are very positive, with more than 80% of follow-up in both consultations, most on a regular basis. On the other hand, when analyzing the follow-up time data, it should also be taken into account that some patients were previously cared for in another centre, either by going to a private centre or because they lived in another healthcare area.

A particularly interesting variable in our study is age. As can be seen, there is a 15-year difference between the median age at the onset of symptoms and the median age at the beginning of follow-up. This seems to condition their chronification, since patients acquire deeply ingrained behaviors for a long time, which makes it difficult to change behaviors with conventional treatments, especially if we take into account that when the follow-up begins, patients present as adults, a circumstance that signals the need for a different approach to that recommended in most studies, which are based on results in adolescent patients [17]. These results also highlight the importance of raising the diagnosis of ED in patients with a typical behaviour of this group of diseases despite not being in adolescence.

The study on complications that arose during follow-up yields several data points to take into account. The first of these is the high proportion of mental comorbidity obtained, close to 75%, a result that is expected given the relationship between ED and personality disorder, mood disorders, obsessive–compulsive disorder and anxiety disorders [18]. There were also high proportions of both a history of suicide behavior disorder and of substance use disorder, both previously described in the scientific literature in relation to the impulsivity that characterizes patients with ED, especially in patients with BN [19]. Furthermore, the consumption of toxins is higher than that estimated in the general population in Spain (5.1%) [20]. On the other hand, among the physical complications, both osteoporosis and hypogonadotropic hypogonadism stand out, both because of their high proportion (40.3% and 22.4% in patients with a median age of 42 years) and because statistically significant differences have been established between the subgroups of AN and BN. This is logical since both are complications related to malnutrition [21] and patients with AN showed significantly lower BMIs than patients with BN. For this same reason, the data obtained regarding Endocrinology admissions makes sense, since such admissions occur when the reason is malnutrition and not decompensation of its mental comorbidity, in which a higher median income was found in the subgroup of AN. Finally, the fact that only one patient has died in this follow-up period is a relatively very positive finding, since a standardized mortality rate of 1.93 has been described in patients with BN and a mortality rate of 4 to 20% in AN, with chronicity being a described risk factor [22].

The limitations of our study are its observational and retrospective nature, the small sample size, and the fact that it was performed in a single centre. The main implications of our study are those mentioned on the age of our patients, as well as the high percentage of physical and mental comorbidities with which they presented. The fact that such a high percentage of patients presented with such comorbidities emphasizes the importance of closely monitoring them, with attention to the prevention and treatment of complications.

## Conclusions

Our sample of patients with SE-ED reveals a high rate of physical complications and mental comorbidity. In the current study, it was found that patients with AN have higher rates of osteoporosis and hypogonadotropic hypogonadism than patients with BN, as well as a lower BMI. Furthermore, our SE-ED patients begin treatment after years of illness, which may condition the prognosis and makes it necessary to search for an early diagnosis, in addition to agreeing on chronicity criteria and raising the need for different therapeutic approaches in these patients.

## Data Availability

The data that support the findings of this study are available from the corresponding author upon reasonable request.

## Abbreviations

SE-ED: Severe and enduring eating disorders
AN: Anorexia nervosa
BN: Bulimia nervosa
SSRI: Selective serotonin reuptake inhibitors
DSM-5: Diagnostic and Statistical Manual of Mental Disorders
BMI: Body mass index
ED: Eating disorders
SPSS®: Statistical Package for Social Science
